# Supplementary figures and images for: External validity of machine learning-based prognostic scores for cystic fibrosis: A retrospective study using the UK and Canadian registries
Source: PLOS Digit Health. 2023 Jan 12;2(1):e0000179. doi: 10.1371/journal.pdig.0000179 (PMC9931238; doi:10.1371/journal.pdig.0000179)

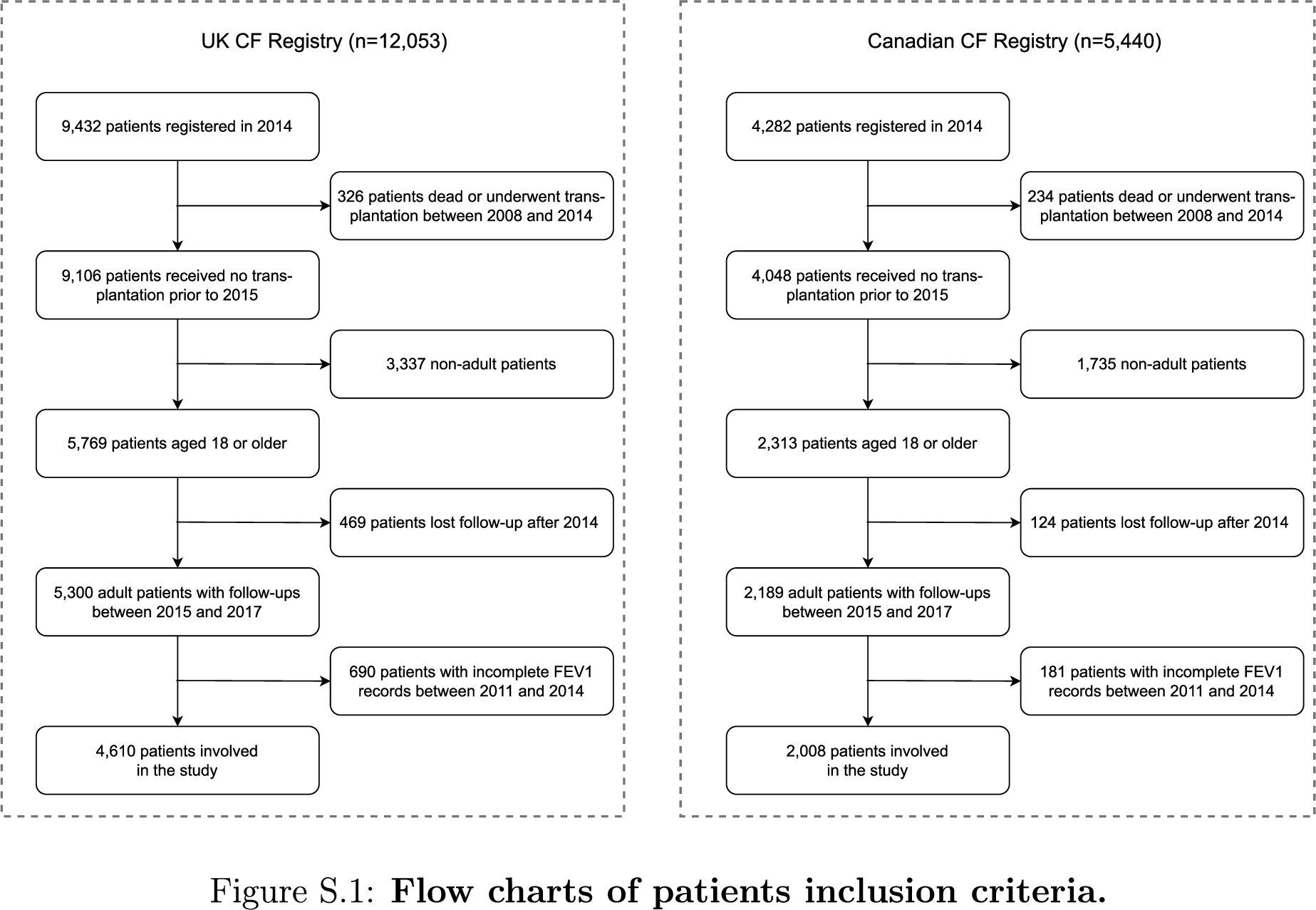

Supplement: S1 Fig — (TIF) [file pdig.0000179.s001.tif]

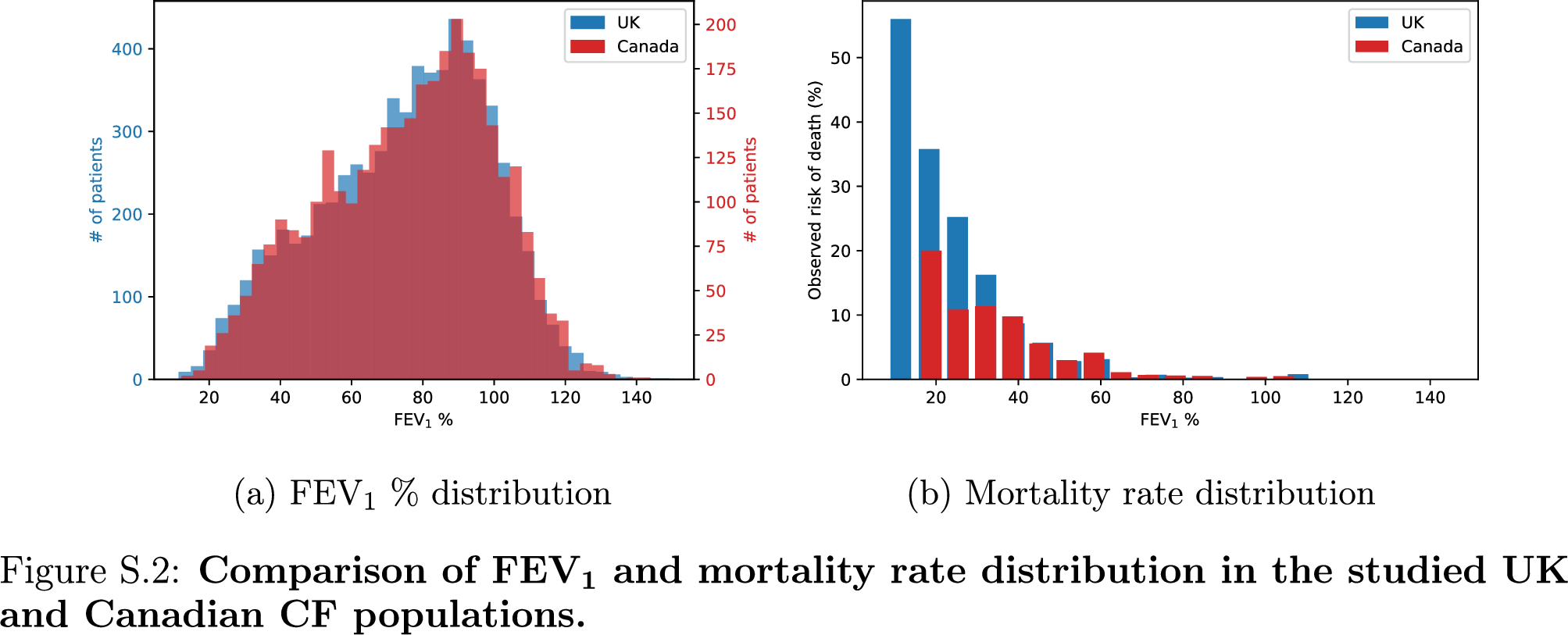

Supplement: S2 Fig — (TIF) [file pdig.0000179.s002.tif]

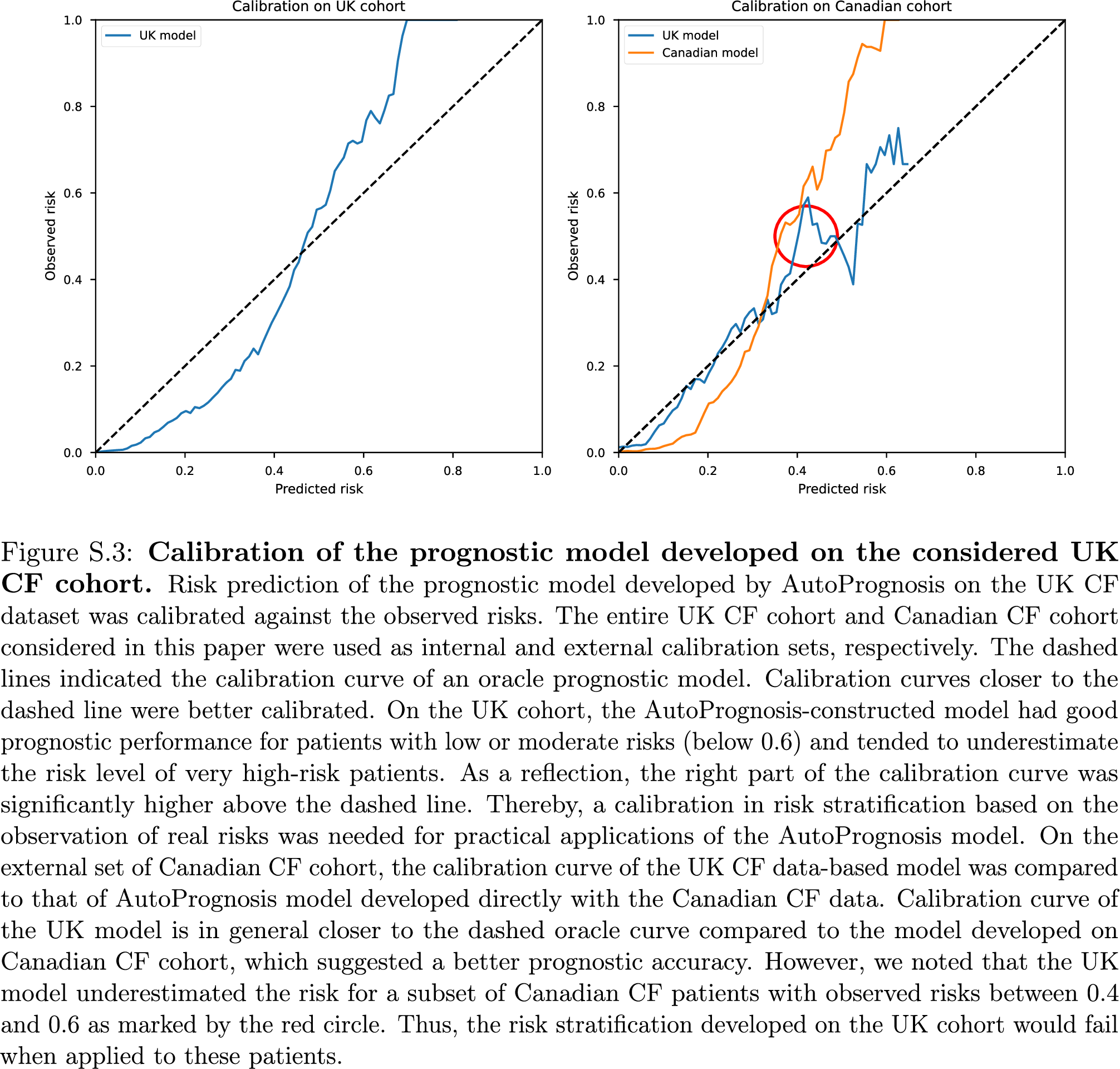

Supplement: S3 Fig — (TIF) [file pdig.0000179.s003.tif]

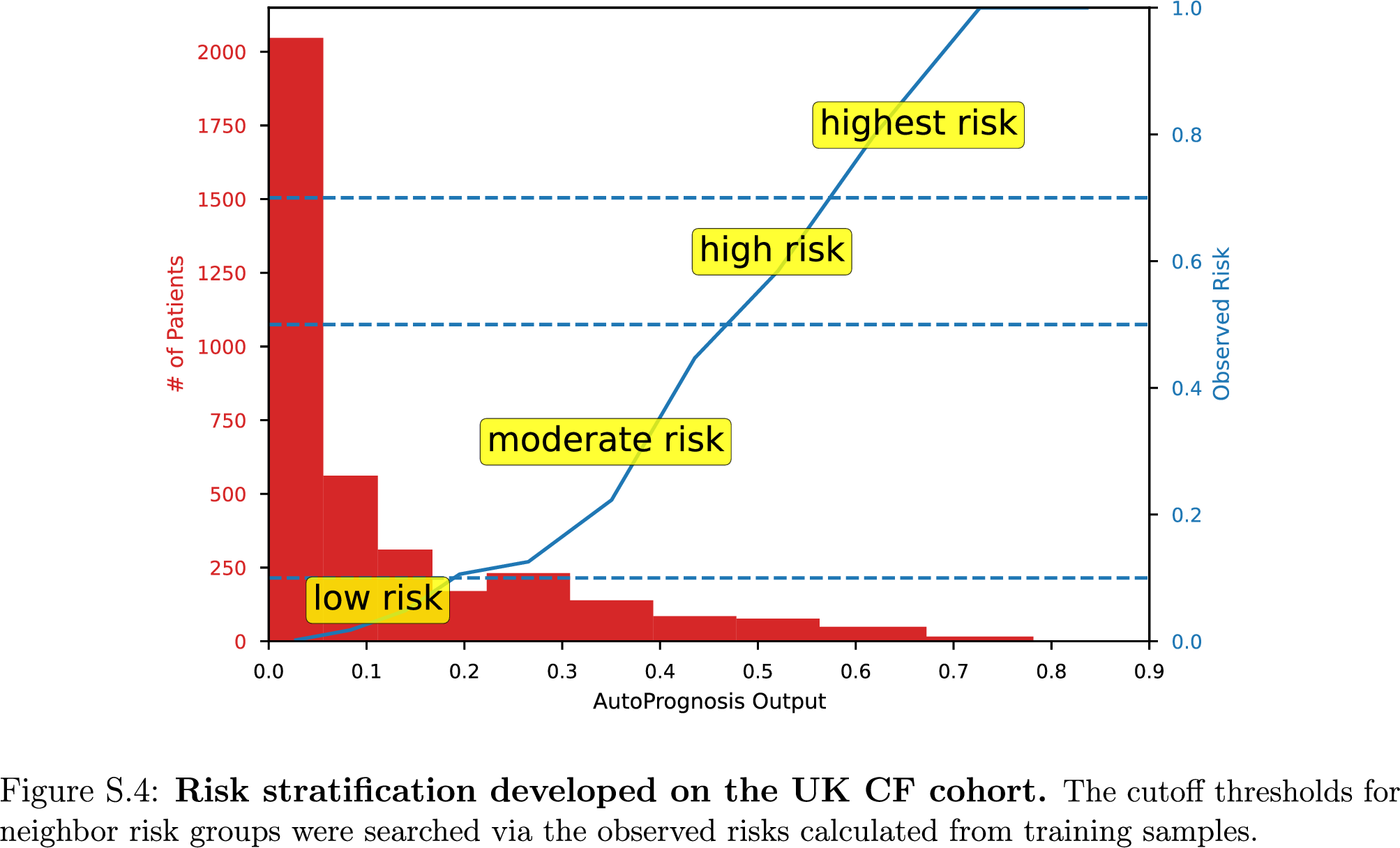

Supplement: S4 Fig — (TIF) [file pdig.0000179.s004.tif]

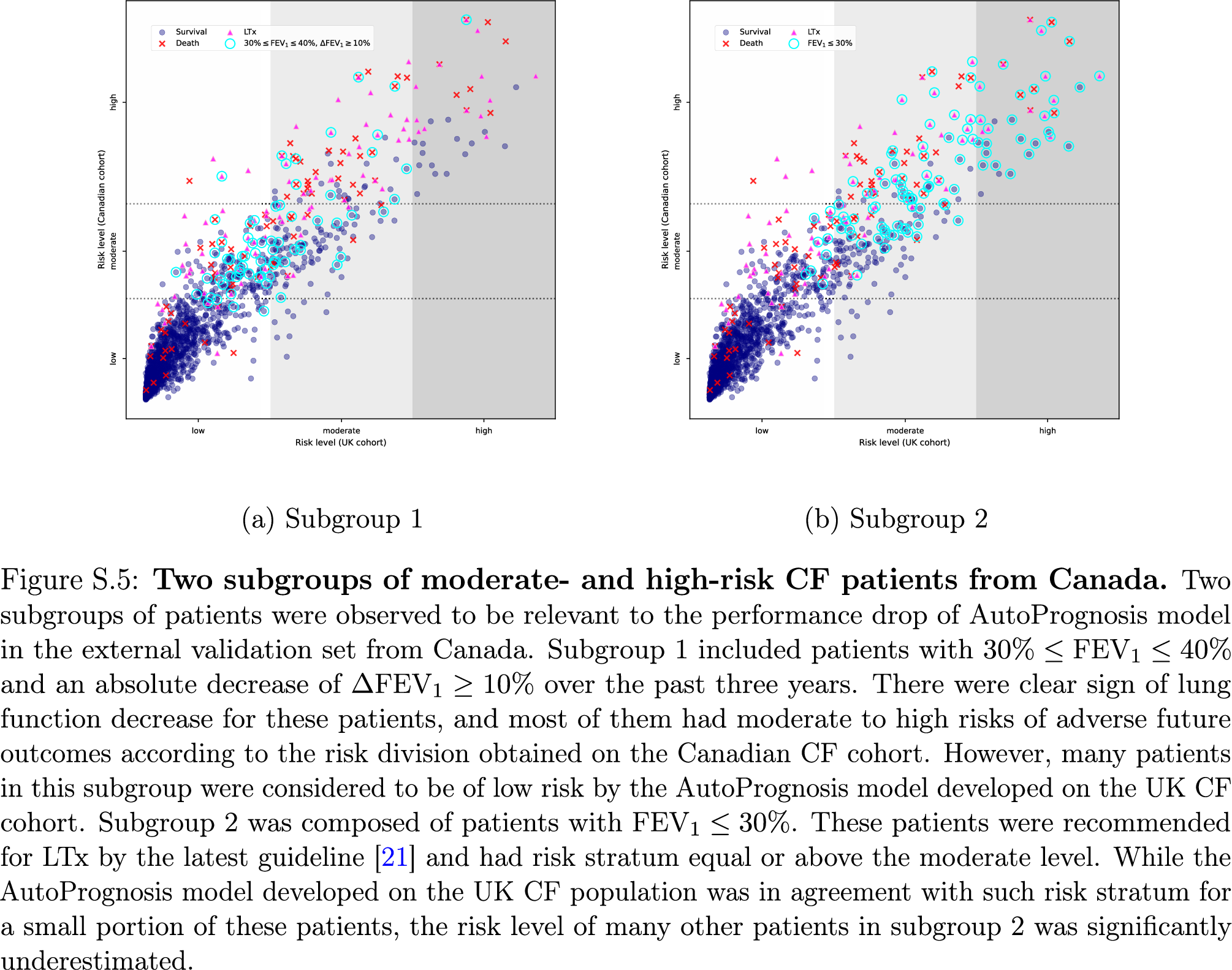

Supplement: S5 Fig — (TIF) [file pdig.0000179.s005.tif]

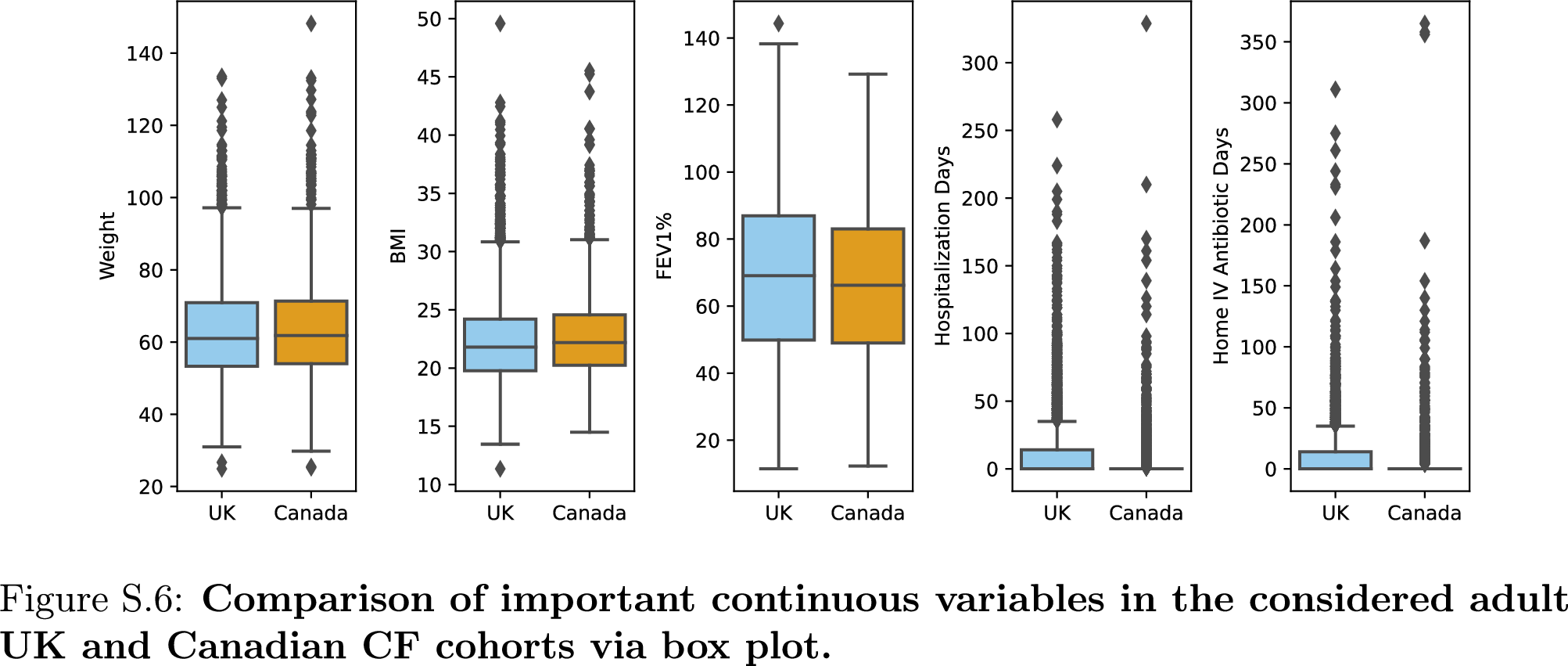

Supplement: S6 Fig — (TIF) [file pdig.0000179.s006.tif]
