# Supplementary material for: External validity of machine learning-based prognostic scores for cystic fibrosis: A retrospective study using the UK and Canadian registries
Source: PLOS Digit Health. 2023 Jan 12;2(1):e0000179. doi: 10.1371/journal.pdig.0000179 (PMC9931238; doi:10.1371/journal.pdig.0000179)
Supplement: S1 Table — (PDF) [file pdig.0000179.s007.pdf]

Table S.1: **LTx rates in subgroups in the studied adult UK and Canadian CF cohorts.** Significant gaps in LTx rate were observed in several subgroups of CF patients in the UK and Canadian CF populations. The proportion of patients received LTx in the next three years was reported together with occurrence and total number of patients in each subgroup.

Subgroup		UK	Canada
Studied cohort	$FEV_1 \leq 30\%$	13.85% (36/260)	32.69% (34/104)
	$FEV_1 > 30\%$	0.97% (42/4350)	2.78% (53/1904)
$30\% \leq FEV_1 \leq 40\%$	$\Delta FEV_1 \geq 10\%$	5.29% (10/189)	21.43% (15/70)
	$\Delta FEV_1 < 10\%$	5.42% (13/240)	9.16% (12/131)
$FEV_1 \leq 30\%$	$BMI \leq 18.5\ kg/m^2$	9.72% (7/72)	27.59% (8/29)
	$BMI > 18.5\ kg/m^2$	15.43% (29/188)	34.67% (26/75)
