# Supplementary material for: External validity of machine learning-based prognostic scores for cystic fibrosis: A retrospective study using the UK and Canadian registries
Source: PLOS Digit Health. 2023 Jan 12;2(1):e0000179. doi: 10.1371/journal.pdig.0000179 (PMC9931238; doi:10.1371/journal.pdig.0000179)
Supplement: S1 Appendix — (PDF) [file pdig.0000179.s008.pdf]

Supplementary material

A.1 Missing data imputation

In both the extracted UK and Canadian CF data, missing values in variables related to treatment, comorbidity, and microbiology were imputed with zeroes. Missing measurements of height, weight, BMI and FEV_1 were forward-filled with last observation, and the average value at population level was used instead when no previous observation for the same patient was available. Other missingness was kept as is in the original data.

A.2 Mutation categories

Based on the five standard CF gene mutation classes [37], in this study, the CF pateints were classified into five categories as follows.

- Class A: mutation class I and class II (other than ΔF508);
- Class B: ΔF508;
- Class C: Ivacaftor responsive mutations;
- Class D: mutation class IV and class V;
- Class O: all other cases.

The Ivacaftor is a type of cystic fibrosis transmembrane conductance regulator (CFTR) modulator. The Ivacaftor responsive genes considered in this study include E56K, G178R, S549R, K1060T, G1244E, P67L, E193K, G551D, A1067T, S1251N, R74W, L206W, G551S, G1069R, S1255P, D110E, R347H, D579G, R1070Q, D1270N, D110H, R352Q, S945L, R1070W, G1349D, R117H, A455E, S977F, F1074L, R117C, S549N, F1052V, and D1152H. Related to mutation categories, the pancreatic insufficiency score was calculated from the CF gene mutations as a reflection of the gene expression level.

A.3 LTx referral policy derived from the 2019 guideline

Based on the 2019 guideline [21] and the feature variables considered in this study, the derived policy recommended LTx for a CF patient in the following cases:

- FEV_1 below 50% predicted and there was a relative decline in FEV_1% predicted above 20% in the past 12 months;
- FEV_1 below 40% predicted with markers of reduced survival time, including BMI below 18 kg/m^2, presence of pneumothorax or hemoptysis, and treatment of IV antibiotics at home [22, 23, 21];
- FEV_1 below 30% predicted.

A.4 Additional statistical analysis of patient characteristics

Complementary to the statistics of patient characteristics in Table 2 of the main manuscript, p-values from analysis of variance (ANOVA) for equal population means and median test for equal population medians are reported in Table A.1 as follows.

Table A.1: **P-values from ANOVA and median test of major characteristics of the studied UK and Canadian CF cohorts in 2014.** Binary variables are marked by *. Small p-value, i.e., $p < 0.05$, indicates that the two populations have different mean or median values.

	Variable	ANOVA	Median Test
Demographics	Age	0.8091	0.2463
	Male*	0.9837	N/A
	Female*	0.9837	N/A
	Height	0.0437	0.3192
	Weight	0.0059	0.0384
	BMI	0.0000	0.0001
	FEV1%	0.0001	0.0021
	Insufficiency Allele	0.0000	0.0000
Treatment	Oxygen Therapy*	0.0000	0.0000
	IV Antibiotic Home*	0.0000	0.0000
	Hospitalization*	0.0000	0.0000
	Ivacaftor*	0.0000	0.0000
	HyperSaline*	0.0000	0.0000
	Inhaled Colistin*	0.0000	0.0000
	Chronic Macrolide*	0.0000	N/A
	Cortico Oral*	0.0000	0.0000
	Cortico Inhaled*	0.0000	0.0000
	Cortico Combo*	0.0000	0.0000
	Antifungals*	0.0000	0.0000
	HDI Buprofen*	0.0047	0.0173
Comorbidity	Liver Cirrhosis*	0.0900	0.1005
	ABPA*	0.0000	0.0000
	Hemoptysis*	0.0000	0.0000
	Pneumothorax*	0.0128	0.0199
	Sinus Disease*	0.0000	0.0000
	Pancreatitis*	0.0463	0.0619
	Intestinal Obstruction*	0.0761	0.0851
	Cancer*	0.0003	0.0006
	Fracture*	0.8723	0.9937
	Bone Loss*	0.0000	0.0000
	Depression/Anxiety*	0.0000	0.0000
Genetics	Mutation Category AB*	0.6688	0.6944
	Mutation Category BB*	0.0001	N/A
	Mutation Category BC*	0.0020	0.0023
	Mutation Category BO*	0.0002	0.0002
Microbiology	Burkholderia Cepacia*	0.3257	0.3578
	Pseudomonas*	0.0000	N/A
	MRSA*	0.0000	0.0000
	Aspergillus*	0.0000	0.0000

A.5 Diagnostic performance on the single endpoint of death

Table A.2: **Diagnostic performance of three LTx referral policies on the single endpoint of death without LTx.**

Prognostic performance		$FEV_1 \leq 30\%$	Guideline 2019 [21]	AutoPrognosis	
				Original	Augmented
Source validation set (UK cohort)	PPV	0.32±0.03	0.21±0.01	0.39±0.04	0.26±0.01
	TPR	0.37±0.05	0.59±0.06	0.35±0.06	0.56±0.04
	F1	0.34±0.04	0.31±0.02	0.36±0.04	0.36±0.01
	AUCROC	–	–	0.89±0.02	–
	AUCPRC	–	–	0.34±0.04	–
External validation set (Canadian cohort)	PPV	0.14±0.00	0.12±0.00	0.16±0.01	0.14±0.00
	TPR	0.21±0.00	0.39±0.00	0.10±0.02	0.34±0.00
	F1	0.17±0.00	0.18±0.00	0.12±0.01	0.19±0.00
	AUCROC	–	–	0.84±0.00	–
	AUCPRC	–	–	0.16±0.00	–

A.6 Major characteristics of two patient subgroups

Table A.3: **Major characteristics of patient subgroup 1 (30% $\leq$ FEV$_1$ $\leq$ 40% with ΔFEV$_1$ $\geq$ 10%) from considered UK and Canadian CF populations in 2014.** Binary variables were marked by * with occurrence and incidence rate reported. Continuous variables were reported with median value and IQR. Variables with different mean values in these two populations were identified via two-sample t-test under the p-value of 0.05 and were marked with †. Binary variables with a gap over 10% in incidence rate between the two populations were highlighted in bold.

	Variable	UK	Canada	p-value
Demographics	Age	30.50 (14.08)	28.93 (14.61)	0.2257
	Male*	84 (44.44%)	38 (54.29%)	0.1600
	Female*	105 (55.56%)	32 (45.71%)	0.1600
	Height	165.20 (13.70)	168.00 (12.49)	0.1750
	Weight	54.00 (13.90)	56.07 (16.31)	0.1114
	BMI	19.52 (3.63)	20.15 (3.70)	0.2762
	FEV1%†	35.01 (5.28)	36.56 (4.50)	0.0394
	Insufficiency Allele	1.96 (0.06)	1.96 (0.00)	0.8677
Treatment	Oxygen Therapy*	42 (22.22%)	9 (12.86%)	0.0930
	**IV Antibiotic Home***†	115 (60.85%)	29 (41.43%)	0.0051
	**Hospitalization***†	144 (76.19%)	36 (51.43%)	0.0001
	Ivacaftor*	6 (3.17%)	3 (4.29%)	0.6660
	HyperSaline*	81 (42.86%)	25 (35.71%)	0.3010
	**Inhaled Colistin***†	104 (55.03%)	0 (0.00%)	0.0000
	Chronic Macrolide*	126 (66.67%)	45 (64.29%)	0.7206
	Cortico Oral*	33 (17.46%)	6 (8.57%)	0.0762
	**Cortico Inhaled***†	35 (18.52%)	5 (7.14%)	0.0244
	**Cortico Combo***†	101 (53.44%)	1 (1.43%)	0.0000
	Antifungals*†	21 (11.11%)	1 (1.43%)	0.0130
	HDI Buprofen*	0 (0.00%)	0 (0.00%)	N/A
Comorbidity	Liver Cirrhosis*	11 (5.82%)	3 (4.29%)	0.6293
	**ABPA***†	31 (16.40%)	1 (1.43%)	0.0011
	**Hemoptysis***†	40 (21.16%)	1 (1.43%)	0.0001
	Pneumothorax*	3 (1.59%)	0 (0.00%)	0.2908
	Sinus Disease*	30 (15.87%)	17 (24.29%)	0.1197
	Pancreatitis*	1 (0.53%)	0 (0.00%)	0.5438
	Intestinal Obstruction*	9 (4.76%)	3 (4.29%)	0.8720
	Cancer*	1 (0.53%)	1 (1.43%)	0.4646
	Fracture*	2 (1.06%)	2 (2.86%)	0.2989
	**Bone Loss***†	53 (28.04%)	8 (11.43%)	0.0050
	**Depression/Anxiety***†	19 (10.05%)	14 (20.00%)	0.0331
Genetics	Mutation Category AB*	41 (21.69%)	11 (15.71%)	0.2879
	Mutation Category BB*	104 (55.03%)	36 (51.43%)	0.6075
	Mutation Category BC*	12 (6.35%)	4 (5.71%)	0.8512
	Mutation Category BO*	12 (6.35%)	9 (12.86%)	0.0890
Microbiology	Burkholderia Cepacia*	16 (8.47%)	2 (2.86%)	0.1159
	**Pseudomonas***†	167 (88.36%)	49 (70.00%)	0.0004
	**MRSA***†	5 (2.65%)	12 (17.14%)	0.0000
	Aspergillus*	45 (23.81%)	17 (24.29%)	0.9367

Table A.4: **Major characteristics of patient subgroup 2 ($FEV_1 \leq 30\%$) from considered UK and Canadian CF populations in 2014.** Binary variables were marked by * with occurrence and incidence rate reported. Continuous variables were reported with median value and IQR. Variables with different mean values in these two populations were identified via two-sample t-test under the p-value of 0.05 and were marked with †. Binary variables with a gap over 10% in incidence rate between the two populations were highlighted in bold.

	Variable	UK	Canada	p-value
Demographics	Age	33.50 (11.79)	30.75 (13.65)	0.0567
	Male*	149 (57.31%)	60 (57.69%)	0.9467
	Female*	111 (42.69%)	44 (42.31%)	0.9467
	Height	166.20 (15.15)	165.00 (12.08)	0.7450
	Weight	55.90 (17.33)	55.67 (14.30)	0.7715
	BMI	19.84 (4.22)	19.95 (4.65)	0.8682
	FEV1%	25.00 (5.37)	25.54 (5.01)	0.2502
	Insufficiency Allele	1.96 (0.06)	1.96 (0.05)	0.5084
Treatment	**Oxygen Therapy***†	126 (48.46%)	37 (35.58%)	0.0255
	**IV Antibiotic Home***†	164 (63.08%)	29 (27.88%)	0.0000
	**Hospitalization***†	191 (73.46%)	48 (46.15%)	0.0000
	Ivacaftor*	13 (5.00%)	3 (2.88%)	0.3752
	HyperSaline*	103 (39.62%)	33 (31.73%)	0.1610
	**Inhaled Colistin***†	155 (59.62%)	1 (0.96%)	0.000
	**Chronic Macrolide***†	195 (75.00%)	61 (58.65%)	0.0020
	**Cortico Oral***†	69 (26.54%)	5 (4.81%)	0.0000
	Cortico Inhaled*	26 (10.00%)	4 (3.85%)	0.0540
	**Cortico Combo***†	153 (58.85%)	1 (0.96%)	0.0000
	Antifungals*†	24 (9.23%)	2 (1.92%)	0.0144
	HDI Buprofen*	0 (0.00%)	1 (0.96%)	0.1140
Comorbidity	Liver Cirrhosis*	21 (8.08%)	8 (7.69%)	0.9029
	**ABPA***†	38 (14.62%)	1 (0.96%)	0.0001
	**Hemoptysis***†	46 (17.69%)	2 (1.92%)	0.0001
	Pneumothorax*	13 (5.00%)	3 (2.88%)	0.3752
	**Sinus Disease***†	26 (10.00%)	25 (24.04%)	0.0005
	Pancreatitis*	1 (0.38%)	0 (0.00%)	0.5278
	Intestinal Obstruction*	24 (9.23%)	4 (3.85%)	0.0820
	Cancer*	0 (0.00%)	1 (0.96%)	0.1140
	Fracture*	3 (1.15%)	2 (1.92%)	0.5702
	**Bone Loss***†	136 (52.31%)	11 (10.58%)	0.0000
	Depression/Anxiety*	33 (12.69%)	18 (17.31%)	0.2530
Genetics	Mutation Category AB*	45 (17.31%)	23 (22.12%)	0.2890
	Mutation Category BB*	148 (56.92%)	50 (48.08%)	0.1265
	Mutation Category BC*	23 (8.85%)	4 (3.85%)	0.1006
	Mutation Category BO*	21 (8.08%)	9 (8.65%)	0.8570
Microbiology	Burkholderia Cepacia*	24 (9.23%)	8 (7.69%)	0.6407
	**Pseudomonas***†	231 (88.85%)	73 (70.19%)	0.0000
	MRSA*	13 (5.00%)	9 (8.65%)	0.1873
	Aspergillus*	54 (20.77%)	28 (26.92%)	0.2053

References

[1] Kapnadak SG, Dimango E, Hadjiliadis D, Hempstead SE, Tallarico E, Pilewski JM, et al. Cystic Fibrosis Foundation consensus guidelines for the care of individuals with advanced cystic fibrosis lung disease. Journal of Cystic Fibrosis. 2020;19(3):344–354.

[2] Saunders T, Burgner D, Ranganathan S. Identifying and preventing cardiovascular disease in patients with cystic fibrosis. Nature Cardiovascular Research. 2022;1(3):187–188.

[3] Yeung JC, Machuca TN, Chaparro C, Cypel M, Stephenson AL, Solomon M, et al. Lung transplantation for cystic fibrosis. The Journal of Heart and Lung Transplantation. 2020;39(6):553–560.

[4] Kerem E, Reisman J, Corey M, Canny GJ, Levison H. Prediction of mortality in patients with cystic fibrosis. New England Journal of Medicine. 1992;326(18):1187–1191.

[5] Yoon J, Davtyan C, van der Schaar M. Discovery and clinical decision support for personalized healthcare. IEEE Journal of Biomedical and Health Informatics. 2016;21(4):1133–1145.

[6] Alaa AM, van der Schaar M. Prognostication and risk factors for cystic fibrosis via automated machine learning. Scientific Reports. 2018;8(1):1–19.

[7] Scotet V, L'Hostis C, Férec C. The changing epidemiology of cystic fibrosis: incidence, survival and impact of the CFTR gene discovery. Genes. 2020;11(6):589.

[8] Bailly A, Blanc C, Francis É, Guillotin T, Jamal F, Wakim B, et al. Effects of dataset size and interactions on the prediction performance of logistic regression and deep learning models. Computer Methods and Programs in Biomedicine. 2022;213:106504.

[9] Alaa AM, Gurdasani D, Harris AL, Rashbass J, van der Schaar M. Machine learning to guide the use of adjuvant therapies for breast cancer. Nature Machine Intelligence. 2021;3(8):716–726.

[10] Alaa A, Schaar M. Autoprognosis: Automated clinical prognostic modeling via bayesian optimization with structured kernel learning. In: International Conference on Machine Learning. Proceedings of Machine Learning Research. 2018:139–148.

[11] Imrie F, Cebere B, McKinney EF, and Schaar M. Autoprognosis 2.0: Democratizing diagnostic and prognostic modeling in healthcare with automated machine learning. arXiv preprint arXiv:2210.12090. 2022.

[12] AutoPrognosis - A System for Automating the Design of Predictive Modeling Pipelines Tailored for Clinical Prognosis; 2022. Available from: https://github.com/vanderschaarlab/autoprognosis.

[13] UK Cystic Fibrosis Registry 2014 Annual Data Report; 2015. Available from: https://www.cysticfibrosis.org.uk/sites/default/files/2020-12/2014 Registry Annual Data Report final.pdf.

[14] The Canadian Cystic Fibrosis Registry 2014 Annual Report; 2016. Available from: https://www.cysticfibrosis.ca/registry/2014AnnualDataReport.pdf.

[15] Cardiothoracic Advisory Group (CTAG) on behalf of NHSBT. Donor lung distribution and allocation.; 2017. Available from: http://odt.nhs.uk/pdf/lung_allocation_policy.pdf.

[16] Kourliouros A, Hogg R, Mehew J, Al-Aloul M, Carby M, Lordan JL, et al. Patient outcomes from time of listing for lung transplantation in the UK: are there disease-specific differences? Thorax. 2019;74(1):60–68.

[17] Hirji A, Zhao H, Ospina MB, Lomelin JS, Halloran K, Hubert M, et al. Clinical judgment versus lung allocation score in predicting lung transplant waitlist mortality. Clinical Transplantation. 2020;34(7):e13870.

[18] McCarthy M. Cystic fibrosis patients in Canada live nearly a decade longer than those in US, study finds. British Medical Journal. 2017;356.

[19] Stephenson AL, Sykes J, Stanojevic S, Quon BS, Marshall BC, Petren K, et al. Survival comparison of patients with cystic fibrosis in Canada and the United States: a population-based cohort study. Annals of Internal Medicine. 2017;166(8):537–546.

[20] International Registry in Organ Donation and Transplantation; 2022. Available from: https://www.irodat.org/?p=database.

[21] Ramos KJ, Smith PJ, McKone EF, Pilewski JM, Lucy A, Hempstead SE, et al. Lung transplant referral for individuals with cystic fibrosis: Cystic Fibrosis Foundation consensus guidelines. Journal of Cystic Fibrosis. 2019;18(3):321–333.

[22] Nkam L, Lambert J, Latouche Al, Bellis G, Burgel PR, Hocine M. A 3-year prognostic score for adults with cystic fibrosis. Journal of Cystic Fibrosis. 2017;16(6):702–708.

[23] Liou TG, Adler FR, FitzSimmons SC, Cahill BC, Hibbs JR, Marshall BC. Predictive 5-year survivorship model of cystic fibrosis. American Journal of Epidemiology. 2001;153(4):345–352.

[24] Greiner M, Pfeiffer D, Smith R. Principles and practical application of the receiver-operating characteristic analysis for diagnostic tests. Preventive Veterinary Medicine. 2000;45(1-2):23–41.

[25] Singer JP, Peterson ER, Snyder ME, Katz PP, Golden JA, D'Ovidio F, et al. Body composition and mortality after adult lung transplantation in the United States. American Journal of Respiratory and Critical Care Medicine. 2014;190(9):1012–1021.

[26] Jennerich AL, Pryor JB, Wai TYH, Kapnadak SG, Aitken ML, Goss CH, et al. Low body mass index as a barrier to lung transplant in cystic fibrosis. Journal of Cystic Fibrosis. 2021;.

[27] Ruttens D, Verleden S, Vandermeulen E, Vos R, Van Raemdonck D, Vanaudenaerde B, et al. Body mass index in lung transplant candidates: a contra-indication to transplant or not? Transplantation Proceedings. 2014;46:1506–1510.

[28] Ramos KJ, Kapnadak SG, Bradford MC, Somayaji R, Morrell ED, Pilewski JM, et al. Underweight patients with cystic fibrosis have acceptable survival following lung transplantation: a united network for organ sharing registry study. Chest. 2020;157(4):898–906.

[29] Aaron SD, Stephenson AL, Cameron DW, Whitmore GA. A statistical model to predict one-year risk of death in patients with cystic fibrosis. Journal of Clinical Epidemiology. 2015;68(11):1336–1345.

[30] Courtney J, Bradley J, Mccaughan J, O'connor T, Shortt C, Bredin C, et al. Predictors of mortality in adults with cystic fibrosis. Pediatric Pulmonology. 2007;42(6):525–532.

[31] Qiu S, Zhong X. Macrolides: a promising pharmacologic therapy for chronic obstructive pulmonary disease. Therapeutic Advances in Respiratory Disease. 2017;11(3):147–155.

[32] Wark PA, Gibson PG. Allergic bronchopulmonary aspergillosis: new concepts of pathogenesis and treatment. Respirology. 2001;6(1):1–7.

[33] Skowronski E, Fitzgerald DA. Life-threatening allergic bronchopulmonary aspergillosis in a well child with cystic fibrosis. The Medical Journal of Australia. 2005;182(9):482–483.

[34] Bachrach LK, Loutit CW, Moss RB, Marcus R. Osteopenia in adults with cystic fibrosis. The American Journal of Medicine. 1994;96(1):27–34.

[35] Gore AP, Kwon SH, Stenbit AE. A roadmap to the brittle bones of cystic fibrosis. Journal of Osteoporosis. 2010;2011.

[36] Zhu MZ, Levvey BJ, McGiffin DC, Snell GI. An intention-to-treat view of lung transplantation for interstitial lung disease: successful strategies to minimize waiting list and posttransplant mortality. Transplantation. 2022;106(1):188–199.

[37] De Boeck K, Zolin A, Cuppens H, Olesen H, Viviani L. The relative frequency of CFTR mutation classes in European patients with cystic fibrosis. Journal of Cystic Fibrosis. 2014;13(4):403–409.
