# Supplementary material for: External validity of machine learning-based prognostic scores for cystic fibrosis: A retrospective study using the UK and Canadian registries
Source: PLOS Digit Health. 2023 Jan 12;2(1):e0000179. doi: 10.1371/journal.pdig.0000179 (PMC9931238; doi:10.1371/journal.pdig.0000179)
Supplement: Attachment — Submitted filename: response_letter.pdf [file pdig.0000179.s009.pdf]

Authors' Response to Reviews of

External Validity of Machine Learning-based Prognostic Scores for Cystic Fibrosis: A Retrospective Study using the UK and Canadian Registries

Yuchao Qin, Ahmed Alaa, Andres Floto, Mihaela van der Schaar
PLOS Digital Health

RC: ***Reviewers' Comment***, AR: Authors' Response, ☐ Manuscript Text

We thank all reviewers for their time in helping us to evaluate our work. Their insightful comments are greatly appreciated and have helped us to further improve our manuscript. These comments are addressed point-by-point below.

1. Reviewer #1

We wish to express our appreciation to reviewer #1 for the in-depth comments which have helped us to further improve the clarity and quality of our manuscript.

1.1. Method Detail and Justification

RC: ***The methods are generally appropriate, although clarification of a few details and a justification for using this particular method of AutoML should be provided.***

AR: After the submission of our manuscript, we decided to release our AutoML framework under the name of "AutoPrognosis 2.0" and make it a publicly available tool for healthcare researchers [19, 20]. To be consistent with the open-source package, the previous method name "Adjutorium" has been replaced with "AutoPrognosis" throughout the manuscript. We have also included further details of the AutoPrognosis 2.0 framework along with justification on our choice of AutoML approach in the Methods Section of the revised manuscript.

Justification for Adopting AutoPrognosis. We have provided explanations for using the AutoML framework AutoPrognosis in this study as follows.

> For the prognostic task considered in this paper, ~~Adjutorium~~AutoPrognosis ~~was~~ 2.0 [19, 20] is used to search for the optimal ~~ML~~ prognostic model as an ensemble of multiple ML pipelines as illustrated in the overview in Fig 1. ~~As an AutoML framework~~AutoPrognosis 2.0 is an enhanced version of the original AutoPrognosis approach proposed in [10]. For the sake of convenience, we omit the version number and refer to AutoPrognosis 2.0 with AutoPrognosis in the following discussion. AutoPrognosis is to date the only open-source AutoML framework tailored for healthcare studies [20] and is validated to outperform existing AutoML methods [10, 19]. The end-to-end design and rich functionalities of the AutoPrognosis framework make it a convenient tool for clinical model development and allow healthcare professionals to take advantage of state-of-the-art AutoML algorithms in their research without the requirement of extensive knowledge on machine learning [19].

Clarification of AutoPrognosis Approach. Additional explanation of the AutoML workflow in the AutoPrognosis framework has been added as follows. For further algorithmic and technical details, please refer to [19, 20].

> Given an input dataset, ~~Adjutorium~~AutoPrognosis automatically constructs ~~performant, end-to-end~~ ML pipelines as combinations of data imputation, preprocessing, and ~~classification plugins based on the input dataset~~prediction modules in an end-to-end fashion. After calibration of the output function, these ML pipelines are combined as an ensemble for the best ~~prediction performance. Bayesian optimization is then adopted to search for~~ prognostic performance. Existing AutoML search algorithms including the default Bayesian optimization method can be utilized by AutoPrognosis to determine the optimal ensemble ~~structure and pipeline parameters.~~ and ML pipeline configurations [19]. Further technical details can be found in the open-source software package [20]. In complementary to the development of optimal prognostic models, AutoPrognosis also provides clinical investigation functionalities to help clinicians further understand the derived prognostic model via explanation and cohort analysis modules. These inspection tools are specifically tailored for healthcare studies and enable clinicians to gain better understanding on feature variables and their impact on ML model predictions [19].

1.2. Further Dataset Description and Analysis

RC: ***Overall, the results are clear, and the comparison for model validation is good, however, more Datasets description is needed, also the balance and the potential bias from the mismatches between the variables of the datasets should be addressed and spotted as mentioned in the discussion section.***

AR: We have provided more description of the two datasets considered in this study in the revised manuscript. Additional statistical comparisons between the two studied CF cohorts have been included in S1 Appendix to evaluate the difference in their variable distributions. The potential mismatch in variable definition and its impact on our analysis are further addressed in the Discussion Section as well.

Additional Dataset Description. Some additional descriptions of the two datasets were provided in the S1 Appendix of the previous version. In the revision, we have moved them into the main manuscript as follows.

> For the three-year outcome prediction task, the latest available records in 2014 covering over 99% of registered CF patients with annual reviews in the UK and Canada were used for experiments. The list of 53 commonly available variables considered in this study can be found in Table 1. The definition of mutation category considered in this study was provided in S1 Appendix. The FEV_1% predicted values from the past three years before 2014 were included to provide additional information on lung function evolution of CF patients. Pediatric patients were excluded from this study due to low incidence rate of adverse endpoints (LTx or death) considered in this study [11,12]. The complete sample selection criteria were illustrated in S1 Fig.
>
> After removal of pediatric patients and samples with missing values, records of 4,610 and 2,008 patients from the UK and Canadian CF datasets were involved in this study, respectively. Ranges of considered feature variables in the two selected CF cohorts from UK and Canada were presented in Table ~~1~~2. Additionally, hospitalized patients in the considered UK CF cohort in 2014 had a median hospital stay of 15 days with the interquartile range (IQR) of 8–32 days. Similar length of hospital stay was observed in the Canadian cohorts with the median of 14 (IQR: 7–29) days. For patients received

IV antibiotics treatment at home, the total days of treatment had a median of 22 (IQR 14–40) days in the UK while the median in Canada was 20 (IQR: 14–35) days. Further details of the two datasets can be found in the corresponding annual reports [11, 12] from the UK and Canadian CF Registries.

We have updated description in the sample selection criteria in S1 Fig to improve its consistency with our data processing procedure as well. Feature variables commonly available in (or can be derived from) the two datasets are listed in Table R1 (new Table 1 in the revised manuscript).

Table R1: **Common feature variables considered in the study.**

Age	Gender
Height	Weight
BMI	FEV_1 (2014)
FEV_1% (2014)	FEV_1% (2013)
FEV_1% (2012)	FEV_1% (2011)
Aspergillus	Burkholderia Cepacia
Methicillin-resistant Staphylococcus aureus (MRSA)	Pseudomonas
Oxygen Therapy	Home IV Antibiotics Days
Hospitalization Days	Ivacaftor
HyperSaline	Inhaled Colistin
Chronic Macrolide	Cortico Oral
Cortico Inhaled	Cortico Combo
Antifungals	High-dose (HD) Ibuprofen
Allergic Bronchopulmonary Aspergillosis (ABPA)	Hemoptysis
Pneumothorax	Sinus Disease
Pancreatitis	Intestinal Obstruction
Cancer	Bone Fracture
Bone Loss	Depression/Anxiety
Liver Cirrhosis	Pancreatic insufficiency
Mutation Category $\{A,B,C,D,O\} \times \{A,B,C,D,O\}$	

Additional Statistical Analysis. In the revised manuscript, Table 2 (Table 1 in the previous version) provides the median and IQR of major patient characteristics in the two CF datasets. The p-value of two-sample t-test is also reported in Table 2 to indicate whether the two studied CF cohorts have different population means ($p < 0.05$) over individual feature. Complementary to this result, p-values from analysis of variance (ANOVA) for equal population means and median test for equal population medians are reported in Table R2 which is also included in the new version of S1 Appendix.

For important continuous variables identified by risk factor analysis in Fig. 2 of the main manuscript, we further provide comparison of their box plots in the two datasets to illustrate the variations in patient feature distribution as shown in Fig. R1 (new S6 Fig. in the revision).

Mismatches in Variable Definition. Most of patient covariates considered in our analysis are shared (with consistent definition) by the two datasets. Six feature variables with potential mismatch in definition are

Table R2: **P-values from ANOVA and median test of major characteristics of the studied UK and Canadian CF cohorts in 2014.** Binary variables are marked by *. Small p-value, i.e., $p < 0.05$, indicates that the two populations have different mean or median values.

	Variable	ANOVA	Median Test
Demographics	Age	0.8091	0.2463
	Male*	0.9837	N/A
	Female*	0.9837	N/A
	Height	0.0437	0.3192
	Weight	0.0059	0.0384
	BMI	0.0000	0.0001
	FEV1%	0.0001	0.0021
	Insufficiency Allele	0.0000	0.0000
Treatment	Oxygen Therapy*	0.0000	0.0000
	IV Antibiotic Home*	0.0000	0.0000
	Hospitalization*	0.0000	0.0000
	Ivacaftor*	0.0000	0.0000
	HyperSaline*	0.0000	0.0000
	Inhaled Colistin*	0.0000	0.0000
	Chronic Macrolide*	0.0000	N/A
	Cortico Oral*	0.0000	0.0000
	Cortico Inhaled*	0.0000	0.0000
	Cortico Combo*	0.0000	0.0000
	Antifungals*	0.0000	0.0000
	HDI Buprofen*	0.0047	0.0173
Comorbidity	Liver Cirrhosis*	0.0900	0.1005
	ABPA*	0.0000	0.0000
	Hemoptysis*	0.0000	0.0000
	Pneumothorax*	0.0128	0.0199
	Sinus Disease*	0.0000	0.0000
	Pancreatitis*	0.0463	0.0619
	Intestinal Obstruction*	0.0761	0.0851
	Cancer*	0.0003	0.0006
	Fracture*	0.8723	0.9937
	Bone Loss*	0.0000	0.0000
	Depression/Anxiety*	0.0000	0.0000
Genetics	Mutation Category AB*	0.6688	0.6944
	Mutation Category BB*	0.0001	N/A
	Mutation Category BC*	0.0020	0.0023
	Mutation Category BO*	0.0002	0.0002
Microbiology	Burkholderia Cepacia*	0.3257	0.3578
	Pseudomonas*	0.0000	N/A
	MRSA*	0.0000	0.0000
	Aspergillus*	0.0000	0.0000

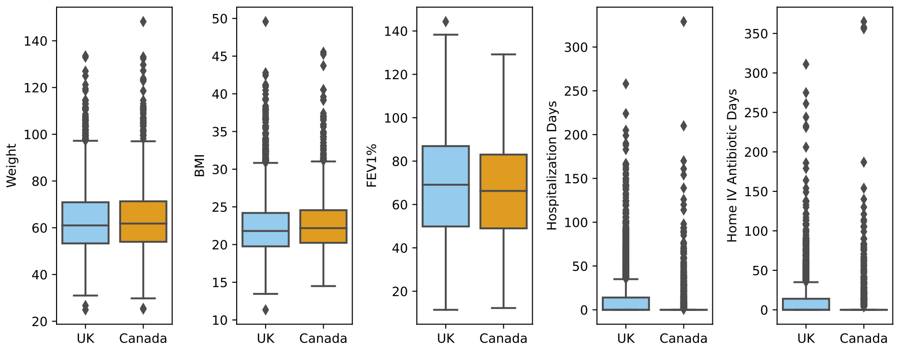

Figure R1: **Comparison of important continuous variables in the considered adult UK and Canadian CF cohorts via box plot.**

listed in Table R3 and are discussed as follows.

Table R3: **Potential mismatches in variable definition.** Six derived variables and their associated variable fields in the UK and Canadian CF datasets are compared below.

Variable	Fields in UK CF dataset	Fields in Canadian CF dataset
Pseudomonas	Intermittent Pseudomonas, Chronic Pseudomonas	Pseudomonas, Multidrug-resistant Pseudomonas (MDRP)
Hemoptysis	Hemoptysis	Massive Hemoptysis
Sinus Disease	Sinus Disease	Nasal Polyps
Bone Fracture	Bone Fracture, Cough Fracture	Bone Fracture
Bone Loss	Osteoporosis, Osteopenia	Osteoporosis
Depression/Anxiety	Depression	Depression, Anxiety

- **Pseudomonas.** The feature variable *pseudomonas* reflects pulmonary infection with pseudomonas aeruginosa (whether intermittent or not). We have combined the “Intermittent Pseudomonas” and “Chronic Pseudomonas” fields from the UK registry and assumed that the combined variable is equivalent to the “MDRP” field and the “Pseudomonas” field recorded in the Canadian CF registry.
- **Haemoptysis.** For the feature variable *haemoptysis*, we have considered “Haemoptysis” variable in the UK data (which will include all degrees of haemoptysis) with “Massive Haemoptysis” variable in the Canadian registry which only refers to severe (massive) haemoptysis and therefore may underrepresent the total number of Canadian patients with any type of haemoptysis.
- **Sinus Disease.** For the feature variable *sinus disease*, we have assumed equivalence of the UK variable “Sinus Disease” (which will include sinusitis, post nasal drip, and nasal polyps) with the Canadian variable “Nasal Polyps” which refers to only nasal polyps and may therefore underrepresent the total number of CF patients in Canada with some sort of sinus disease.
- **Bone Fracture.** For the feature variable *bone fracture*, we have assumed equivalence between the combined UK features of “Bone Fracture” and “Cough Fracture” with the Canadian feature of “Bone Fracture”. This is justified as both sets will capture all bone fractures.

- **Bone Loss.** For the feature variable on *bone loss*, the combined UK variables of "Osteoporosis" and "Osteopenia" will capture all patients with low bone mineral density. The Canadian variable "Osteoporosis" may (but not definitely) only capture patients with bone mineral density z scores less than 2.5, potentially leading to lower patient number.

- **Depression/Anxiety.** For the feature *depression/anxiety*, the UK variable "Depression" is likely to capture both anxiety and depression (usually but not always co-existent in this patient group). The combined Canadian variables "Depression" and "Anxiety" will capture all depression and anxiety. There is therefore a risk that the UK data will miss some patients with isolated anxiety.

None of the mismatches in these feature variables would significantly influence clinicians' estimation of lung transplant or death in CF patients. In the meantime, we would like to emphasize that the mismatch (and the associated bias in a prognostic model on external validation set) itself is one of the focuses of our study. The mismatch in variable definition is only relevant for cross-population application of a prognostic model. The potential bias in ML model prediction on the external validation set (Canadian CF dataset) is related to multiple factors including shifts in patient feature distribution, variations in donor organ resource, differences in lung transplant referral policies and healthcare systems as well as the mismatches in variable definition discussed above. Our study shows that AutoPrognosis can be used to effectively identify the feature variables that contribute to the cross-population bias (e.g., the risk factor analysis in Fig. 2), which allows clinicians to further investigate the influence of different sources of such type of bias. Following the above discussion, we have updated the relevant paragraph in Discussion Section as follows.

> Our study ~~was~~is based on the data obtained from the UK and Canadian Cystic Fibrosis Registries. While we ~~had~~have worked hard to remove possible biases from data processing procedures, the results and conclusion in this paper may be affected by potential ~~mismatches in variable definition or~~ errors in records in these two datasets. In the meantime, mismatches in variable definition across healthcare systems are usually inevitable. Their impact on prognostic biases of an ML model applied across populations could be entangled with other major factors like patient health status and organ availability analyzed in this manuscript. Risk factor analysis in this study shows that variables affected by such type of mismatches have little impact on the external validity of ML models in risk prognostication for CF patients. The impact attribution of different sources of prognostic biases across populations is an important topic, and we consider it as another possible direction of our future work.

In addition, to better align the two datasets, we have excluded the field of "Bilevel Positive Airway Pressure" (BiPAP) while calculating the variable of "Oxygen Therapy" for the Canadian CF dataset in the revised manuscript. The experiment results are updated accordingly.

2. Reviewer #2

RC: ***I should say I was impressed with the quality of the manuscript. Authors have presented a rigorous study supported by extensive statistical analysis and impressive plots. I have no comments and I would recommend it for publication without any hesitation.***

AR: We thank Reviewer #2 for the recognition of our work. Your encouraging comment is greatly appreciated.

3. Minor Changes

Method Name. After the submission of our manuscript, we decided to release our AutoML framework as an open-source software package under the name of “AutoPrognosis 2.0” [19, 20]. We have replaced the previous method name “Adjutorium” with “AutoPrognosis” throughout our manuscript to make it consistent with this change. Two related citations, i.e., [19, 20], have been added to the references of this manuscript.

Reproducibility. To ensure the reproducibility of our results, we have reconducted all experiments with explicitly seeded random number generators. There are very minor changes in numbers reported in the manuscript, which has no significant impact on our conclusion.

Citation Style. We have updated the format of a few references to ensure a consistent citation style.

References

[1] Kapnadak SG, Dimango E, Hadjiliadis D, Hempstead SE, Tallarico E, Pilewski JM, et al. Cystic Fibrosis Foundation consensus guidelines for the care of individuals with advanced cystic fibrosis lung disease. Journal of Cystic Fibrosis. 2020;19(3):344–354.

[2] Saunders T, Burgner D, Ranganathan S. Identifying and preventing cardiovascular disease in patients with cystic fibrosis. Nature Cardiovascular Research. 2022;1(3):187–188.

[3] Yeung JC, Machuca TN, Chaparro C, Cypel M, Stephenson AL, Solomon M, et al. Lung transplantation for cystic fibrosis. The Journal of Heart and Lung Transplantation. 2020;39(6):553–560.

[4] Kerem E, Reisman J, Corey M, Canny GJ, Levison H. Prediction of mortality in patients with cystic fibrosis. New England Journal of Medicine. 1992;326(18):1187–1191.

[5] Yoon J, Davtyan C, van der Schaar M. Discovery and clinical decision support for personalized healthcare. IEEE Journal of Biomedical and Health Informatics. 2016;21(4):1133–1145.

[6] Alaa AM, van der Schaar M. Prognostication and risk factors for cystic fibrosis via automated machine learning. Scientific Reports. 2018;8(1):1–19.

[7] Scotet V, L’Hostis C, Férec C. The changing epidemiology of cystic fibrosis: incidence, survival and impact of the CFTR gene discovery. Genes. 2020;11(6):589.

[8] Bailly A, Blanc C, Francis É, Guillotin T, Jamal F, Wakim B, et al. Effects of dataset size and interactions on the prediction performance of logistic regression and deep learning models. Computer Methods and Programs in Biomedicine. 2022;213:106504.

[9] Alaa AM, Gurdasani D, Harris AL, Rashbass J, van der Schaar M. Machine learning to guide the use of adjuvant therapies for breast cancer. Nature Machine Intelligence. 2021;3(8):716–726.

[10] Alaa A, Schaar M. Autoprognosis: Automated clinical prognostic modeling via bayesian optimization with structured kernel learning. In: International Conference on Machine Learning. Proceedings of Machine Learning Research. 2018:139–148.

[11] UK Cystic Fibrosis Registry 2014 Annual Data Report; 2015. Available from: https://www.cysticfibrosis.org.uk/sites/default/files/2020-12/2014RegistryAnnualDataReportfinal.pdf.

[12] The Canadian Cystic Fibrosis Registry 2014 Annual Report; 2016. Available from: https://www.cysticfibrosis.ca/registry/2014AnnualDataReport.pdf.

[13] Cardiothoracic Advisory Group (CTAG) on behalf of NHSBT. Donor lung distribution and allocation.; 2017. Available from: http://odt.nhs.uk/pdf/lung_allocation_policy.pdf.

[14] Kourliouros A, Hogg R, Mehew J, Al-Aloul M, Carby M, Lordan JL, et al. Patient outcomes from time of listing for lung transplantation in the UK: are there disease-specific differences? Thorax. 2019;74(1):60–68.

[15] Hirji A, Zhao H, Ospina MB, Lomelin JS, Halloran K, Hubert M, et al. Clinical judgment versus lung allocation score in predicting lung transplant waitlist mortality. Clinical Transplantation. 2020;34(7):e13870.

[16] McCarthy M. Cystic fibrosis patients in Canada live nearly a decade longer than those in US, study finds. British Medical Journal. 2017;356.

[17] Stephenson AL, Sykes J, Stanojevic S, Quon BS, Marshall BC, Petren K, et al. Survival comparison of patients with cystic fibrosis in Canada and the United States: a population-based cohort study. Annals of Internal Medicine. 2017;166(8):537–546.

[18] International Registry in Organ Donation and Transplantation; 2022. Available from: https://www.irodat.org/?p=database.

[19] Imrie F, Cebere B, McKinney EF, and Schaar M. Autoprognosis 2.0: Democratizing diagnostic and prognostic modeling in healthcare with automated machine learning. arXiv preprint arXiv:2210.12090. 2022.

[20] AutoPrognosis - A System for Automating the Design of Predictive Modeling Pipelines Tailored for Clinical Prognosis; 2022. Available from: https://github.com/vanderschaarlab/autoprognosis.

[21] Ramos KJ, Smith PJ, McKone EF, Pilewski JM, Lucy A, Hempstead SE, et al. Lung transplant referral for individuals with cystic fibrosis: Cystic Fibrosis Foundation consensus guidelines. Journal of Cystic Fibrosis. 2019;18(3):321–333.

[22] Nkam L, Lambert J, Latouche Al, Bellis G, Burgel PR, Hocine M. A 3-year prognostic score for adults with cystic fibrosis. Journal of Cystic Fibrosis. 2017;16(6):702–708.

[23] Liou TG, Adler FR, FitzSimmons SC, Cahill BC, Hibbs JR, Marshall BC. Predictive 5-year survivorship model of cystic fibrosis. American Journal of Epidemiology. 2001;153(4):345–352.

[24] Greiner M, Pfeiffer D, Smith R. Principles and practical application of the receiver-operating characteristic analysis for diagnostic tests. Preventive Veterinary Medicine. 2000;45(1-2):23–41.

[25] Singer JP, Peterson ER, Snyder ME, Katz PP, Golden JA, D'Ovidio F, et al. Body composition and mortality after adult lung transplantation in the United States. American Journal of Respiratory and Critical Care Medicine. 2014;190(9):1012–1021.

[26] Jennerich AL, Pryor JB, Wai TYH, Kapnadak SG, Aitken ML, Goss CH, et al. Low body mass index as a barrier to lung transplant in cystic fibrosis. Journal of Cystic Fibrosis. 2021;.

[27] Ruttens D, Verleden S, Vandermeulen E, Vos R, Van Raemdonck D, Vanaudenaerde B, et al. Body mass index in lung transplant candidates: a contra-indication to transplant or not? Transplantation Proceedings. 2014;46:1506–1510.

[28] Ramos KJ, Kapnadak SG, Bradford MC, Somayaji R, Morrell ED, Pilewski JM, et al. Underweight patients with cystic fibrosis have acceptable survival following lung transplantation: a united network for organ sharing registry study. Chest. 2020;157(4):898–906.

[29] Aaron SD, Stephenson AL, Cameron DW, Whitmore GA. A statistical model to predict one-year risk of death in patients with cystic fibrosis. Journal of Clinical Epidemiology. 2015;68(11):1336–1345.

[30] Courtney J, Bradley J, Mccaughan J, O'connor T, Shortt C, Bredin C, et al. Predictors of mortality in adults with cystic fibrosis. Pediatric Pulmonology. 2007;42(6):525–532.

[31] Qiu S, Zhong X. Macrolides: a promising pharmacologic therapy for chronic obstructive pulmonary disease. Therapeutic Advances in Respiratory Disease. 2017;11(3):147–155.

[32] Wark PA, Gibson PG. Allergic bronchopulmonary aspergillosis: new concepts of pathogenesis and treatment. Respirology. 2001;6(1):1–7.

[33] Skowronski E, Fitzgerald DA. Life-threatening allergic bronchopulmonary aspergillosis in a well child with cystic fibrosis. The Medical Journal of Australia. 2005;182(9):482–483.

[34] Bachrach LK, Loutit CW, Moss RB, Marcus R. Osteopenia in adults with cystic fibrosis. The American Journal of Medicine. 1994;96(1):27–34.

[35] Gore AP, Kwon SH, Stenbit AE. A roadmap to the brittle bones of cystic fibrosis. Journal of Osteoporosis. 2010;2011.

[36] Zhu MZ, Levvey BJ, McGiffin DC, Snell GI. An intention-to-treat view of lung transplantation for interstitial lung disease: successful strategies to minimize waiting list and posttransplant mortality. Transplantation. 2022;106(1):188–199.

[37] De Boeck K, Zolin A, Cuppens H, Olesen H, Viviani L. The relative frequency of CFTR mutation classes in European patients with cystic fibrosis. Journal of Cystic Fibrosis. 2014;13(4):403–409.
